# Chagas Disease Maternal Seroprevalence and Maternal–Fetal Health Outcomes in a Parturition Cohort in Western El Salvador

**DOI:** 10.3390/tropicalmed8040233

**Published:** 2023-04-20

**Authors:** Mary K. Lynn, Marvin Stanley Rodriguez Aquino, Pamela Michelle Cornejo Rivas, Mufaro Kanyangarara, Stella C. W. Self, Berry A. Campbell, Melissa S. Nolan

**Affiliations:** 1 Department of Epidemiology and Biostatistics, Arnold School of Public Health, University of South Carolina, Columbia, SC 29208, USA; lynnmk@mailbox.sc.edu (M.K.L.); mufaro@mailbox.sc.edu (M.K.); scwatson@mailbox.sc.edu (S.C.W.S.); 2 Health Research and Development Center (CENSALUD), University of El Salvador, San Salvador, El Salvador; marvin.rodriguez@ues.edu.sv (M.S.R.A.); pampdc@hotmail.com (P.M.C.R.); 3 Department of Obstetrics and Gynecology, Prisma Health, Columbia, SC 29203, USA; berry.campbell@prismahealth.org

**Keywords:** Chagas disease, *Trypanosoma cruzi*, vertical transmission, congenital, El Salvador, neglected tropical disease, perinatal, maternal–child health

## Abstract

Congenital Chagas disease is a growing concern, prioritized by the World Health Organization for public health action. El Salvador is home to some of the highest Chagas disease (*Trypanosoma cruzi* infection) burdens in the Americas, yet pregnancy screening remains neglected. This pilot investigation performed a maternal *T. cruzi* surveillance study in Western El Salvador among women presenting for labor and delivery. From 198 consented and enrolled pregnant women, 6% were *T. cruzi* positive by serology or molecular diagnosis. Half of the infants born to *T. cruzi*-positive women were admitted to the NICU for neonatal complications. Geospatial statistical clustering of cases was noted in the municipality of Jujutla. Older women and those knowing an infected relative or close friend were significantly more likely to test positive for *T. cruzi* infection at the time of parturition. In closing, maternal *T. cruzi* infections were significantly higher than national HIV or syphilis maternal rates, creating an urgent need to add *T. cruzi* to mandatory pregnancy screening programs.

## 1. Introduction

Chagas disease, caused by infection with the protozoan parasite *Trypanosoma cruzi,* continues to be the leading cause of non-ischemic cardiomyopathy and a public health concern in the Americas [1,2]. Most infected individuals acquire infection in childhood and experience subclinical chronic disease, in which 30% will insidiously progress to dilated cardiomyopathy and heart failure years to decades after initial infection [3]. An estimated 1 million infected women of child-bearing age are unknowingly infected, posing a considerable risk for both their health and the health of their neonates [4]. Vertical transmission of *T. cruzi* occurs in approximately 5% of infected pregnant women, and up to 60% of these vertically infected infants are asymptomatic at the time of delivery [5,6]. In addition, an estimated 15,000 global cases of congenital infection occur each year with 9000 of these in Latin America [7].

Congenital Chagas disease has been prioritized for public health action by the World Health Organization and is of further clinical importance due to the high efficacy rate of antiparasitic treatment in children under one year of age [8]. Currently, trypanocidal treatment is not recommended for pregnant women, leaving post-infection treatment of newborns, rather than prevention, the main strategy. Congenital transmission routes are poorly understood on a global scale due to the lack of Chagas disease clinical management in pregnant women, universal screening protocols, and prognostic risk factors leading to successful vertical transmission. Early identification of vertical transmission cases is of further importance due to the silent progression of chronic disease that starts in childhood, as cases of heart transplantation and fatality in infants or young adults have been reported [9,10]. Sadly, there is a paucity of research regarding vertical transmission in Central America, leaving both the Chagas disease burden among women of childbearing age and the rate of congenital transmission still largely unknown [2].

The Republic of El Salvador has historically experienced the highest incidence of acute and chronic Chagas disease in Central America, and TcI discrete typing unit is predominant in this country [8,11,12,13,14,15]. Despite great strides toward vector control with the removal of *Rhodnius prolixus* from El Salvador in 2009 [16], vector transmission continues to occur and efforts to reduce human–vector contact in parts of the region have proven difficult. Recent evidence indicates that *Triatoma dimidiata,* the current primary vector causing Chagas disease in Central America, is particularly capable of re-infesting homes shortly after insecticides have been applied, and that control strategies with indoor residual insecticide are not effective in the long term [17]. Since 2016, a high pediatric Chagas disease case burden has occurred in the western department of Sonsonate [18]. Further epidemiologic surveillance of confirmed cases between 2000 and 2012 identified 50% of all cases occurred in children under nine [11]. Our previous work with collaborators at the University of El Salvador and the Ministry of Health in the same department have demonstrated a seroprevalence of 2.3% in cases of pediatric disease [19]. Although transmission dynamics of these cases are unclear, the possibility of congenital cases in this region of high contemporary burden does exist.

Little is known about the prevalence of Chagas disease in pregnant women in El Salvador [8,20]. Few studies have attempted to clarify this burden. Two professional degree theses from 2012 were obtained from the University of El Salvador’s Academic Repository that found 8.6% (10/116) and 1.9% (1/52) seropositivity among pregnant women in the Eastern Region [21,22]. An additional peer-reviewed article regarding serosurveillance of women in the Western Region found 3.8% seroprevalence among 943 pregnant women and 1 vertically infected infant [2]. This 2015 case was the first confirmed congenital infection in El Salvador to date [2]. Despite these findings, Chagas disease remains a neglected disease in El Salvador, with little resources provided to high-burdened health departments to identify and treat cases. Coupled with the extensive history of a high Chagas disease burden in this country, these findings highlight the need for increased research capacity surrounding congenital Chagas disease in El Salvador [2]. Therefore, the goal of the current study was to perform serosurveillance of perinatal women and save biological samples from a well characterized cohort for prospective molecular pathogenesis investigations. Here, we present the seropositive perinatal mothers, their epidemiologic risk factors, and their neonate’s health outcomes noted at birth.

## 2. Materials and Methods

Ethics Statement: This project was approved by the Commité Nacional de Ética de la Investigación en Salud (National Ethics Committee of Health Research) of El Salvador and the Hospital Nacional General “Dr. Jorge Mazzini Villacorta” (Hospital Mazzini). This project was carried out by the University of El Salvador and the University of South Carolina in conjunction with the Salvadoran Ministry of Health and Hospital Mazzini.

Inclusion/Exclusion Criteria: Women of childbearing age were recruited within the Labor and Delivery Department at Hospital Mazzini to participate in this study. Any woman ≥15 years of age with uniparous, non-eclamptic pregnancies, who were physically and mentally able to provide written informed consent, were enrolled to be part of this study. For minors of age <18 years, both written informed assent of the participant and parental/guardian consent was obtained for participation. Women with multiple pregnancies (e.g., pregnant with twins or triplets) or those with previously diagnosed Chagas disease were excluded. Any woman otherwise eligible, that for any reason was deemed unable to participate by the attending physician, was excluded from the study.

Sample collection and screening protocols: A trained nurse from the study site explained study details to all women meeting the inclusion criteria presenting to the Department of Labor and Delivery and obtained written informed consent from all interested women. Consenting women donated a small blood sample ≤20 mL in serum-separator (SST) and ethylenediaminetetraacetic acid disodium salt dihydrate (EDTA) tubes at the time of study enrollment. Each woman was evaluated for pre-eclampsia indicators by blood pressure and urine sample biochemical test strip (Combur-Test strip, Roche, Rotkreuz, Switzerland). Any woman with glycosuria and proteinuria with out-of-range values and blood pressure higher than 130/80 mmHg was defined as at high risk of developing preeclampsia for the purposes of this study. Per standard Chagas disease congenital diagnostic guidelines [23], neonates were not tested at birth and their follow-up nine-month serology status was not available for the current manuscript.

Questionnaires: Enrolled study participants (or a corresponding partner or family member) completed a detailed survey of the participant’s lifetime exposure risk to the triatomine insect vector and other potential *T. cruzi* transmission sources, including tissue or organ transplant. Surveys also contained questions related to the following themes: (1) current pregnancy, demographics, and clinical history (name, age, weight, address, gravidity, parity, current or prior pregnancy complications, comorbidities, symptoms consistent with Chagas disease infection, food security, number of children); (2) description of the family and home (ages of children in the home, prior infections experienced by a member of the family, structure and material of the home, quality of the home structure); (3) vectors (insects typically seen in or around the home, any insect bites in the home and potential adverse reactions, animals kept in or around the home, use of preventive measures such as insecticidal bed nets and indoor residual spraying); and (4) labor, delivery, and neonatal outcomes (gestational age, sex, weight of the neonate, APGAR scores-1 min, 5 min, labor and delivery complications, natural versus Caesarian section (c-section) birth). The full study questionnaire may be found in Supplementary file.

Sample Storage: Samples were stored in a −20 °C freezer and kept in a designated study activity room inside the Labor and Delivery Department in Hospital Mazzini. The study activity room was regularly sanitized to minimize the possibility of sample contamination. Serum-separator tubes were allowed to clot for approximately 30 min and centrifuged within an hour of collection at 1200 RCF g for 10 min. Venous blood tubes were aliquoted on site into 2 mL microcentrifuge tubes with equal parts DNA/RNA Shield (Zymo Research Corporation, Irvine, CA, USA) to ensure sample quality maintenance. Samples were transported weekly in a cooler with ice packs by a study team member to the CENSALUD laboratory at the University of El Salvador, San Salvador, El Salvador. Samples were stored at −80 °C until they were serologically tested in batches of approximately 90 women. Study participants were notified of their results by the study team upon receiving 2 or more positive test results from the three tests performed. The attending OBGYN and Ministry of Health were informed of study test results to initiate Ministry of Health confirmation testing and anti-parasitic treatment of all serologically positive postpartum women.

Serologic and Molecular Methods: *T. cruzi* antibody testing was performed on collected serums samples with the enzyme-linked immunosorbent assays (ELISA) Hemagen Chagas’ Kit (Hemagen Diagnostics, Inc., Columbia, MD, USA), Weiner Chagatest v. 3.0 (Wiener Lab, Rosario, Argentina), and immunochromatographic assay Chagas Stat-Pak (Chembio, Medford, NY, USA). All serologic methods examined the presence of maternal IgG for *T. cruzi*. All samples were tested by the study team using (1) Weiner Chagatest v. 3.0, (2) Hemagen Chagas’ Kit, and (3) Chagas Stat-Pak.

For molecular detection of *T. cruzi* parasite, DNA was extracted from whole blood samples preserved in DNA/RNA Shield using QIAamp 96 Virus QIAcube HT Kit for DNA and RNA (Qiagen, Germantown, MD, USA). Presence of parasite DNA and quantification of parasite load was performed using a next-generation digital polymerase chain reaction (dPCR) assay on the QIAcuity Four Digital PCR system (Qiagen, Germantown, MD, USA) to allow for absolute quantification in each human sample using the previously validated [24,25,26] primers and probes targeting the satellite sequence of the nuclear protozoan genome. Specific sequence information can be found in Supplementary Table S1. Human Rnase P (HsRPP) was used as an internal human control. Chagas disease-positive cases were defined as those with ≥2 positive test serologic test results, as per standard practice [3], or dPCR result of ≥2 partitions. A dPCR result of one partition was considered negative due to the inability to rule out aberrant background signal noise.

Statistical Analysis: Univariate analysis was conducted using Fisher’s exact test to determine associations between Chagas disease status and (1) maternal and neonatal health outcomes and (2) infection risk factors. Multivariable logistic regression was used to determine statistically significant associations and odds ratios for the relationship between Chagas disease and lifetime infection risk factors. Backward stepwise deletion identified a final list of significant covariates in RStudio version 4.1.1 (RStudio, PBC, Boston, MA, USA). Any predictor variable with a variance inflation factor (VIF) ≥7 was excluded from the final model.

Chloropleth maps were created using graduated colors to express positivity by municipality. A Cluster and Outlier Analysis using Anselin Local Moran’s I was conducted to detect spatial autocorrelation and determine hot spots, cold spots, and statistical geospatial outliers for maternal Chagas disease infection. To account for differences in participant recruitment across municipalities, the proportion of positive cases out of the total number of individuals tested per municipality was used in this analysis. Spatial analysis was performed using ArcGIS Pro (Version 2.4, Esri Inc., Redlands, CA, USA).

## 3. Results

**Figure 1:**
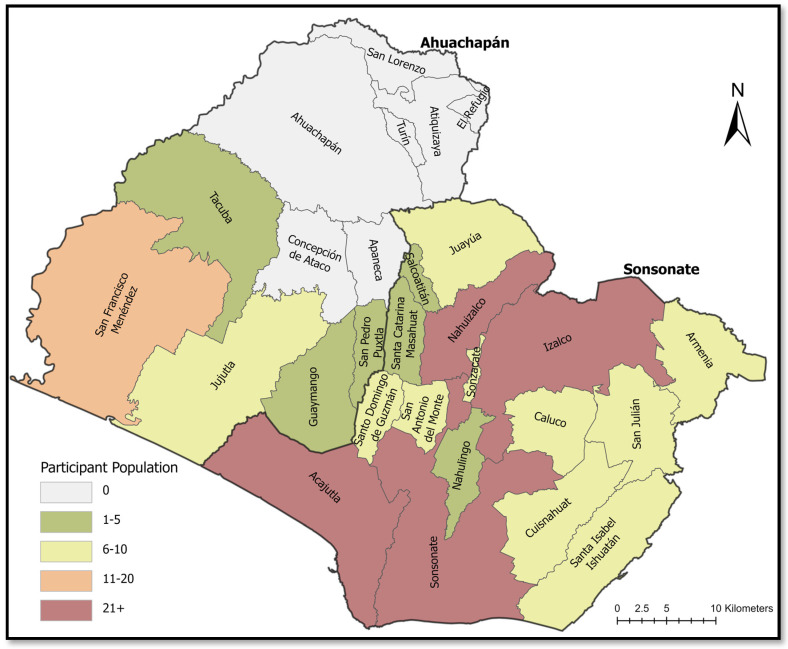
Recruited perinatal Salvadoran women represented all 16 of the Sonsonate department (**right**) municipalities, while 5 municipalities from the equally underserved neighboring Ahuachapan department were also represented (**left**).

From March through September 2022, we enrolled 198 women presenting to the Labor and Delivery Department of Hospital Mazzini. Approximately 84% of women came from Sonsonate department, and the remaining participants derived from the neighboring department, Ahuachapan. Within municipalities, the majority of participants were from nearby Izalco (*n* = 26), Sonsonate (*n* = 23), and Acajutla (*n* = 19) (Figure 1). Participant demographics and clinical history are described in Table 1. The majority of participants were between 18 and 25 years of age and had less than a 7th grade education. Most had one to three living children and 11% reported at least one prior miscarriage. Nearly 96% of all the women in our study had received prenatal care from a health officer in all three trimesters of pregnancy, and none had indicators of preeclampsia at the time of the interview. Other relevant clinical histories present were gestational diabetes (2.0%) and history of seizure (1.0%). The majority of participants in this study lived in very low socioeconomic conditions, with nearly 30% having at least one of the following significant substandard housing indicators: barren earth floor, no electricity, or no potable water source in the home. Over half of participants had outdoor latrine access only (5.6%). Prior negative obstetric or fetal health outcomes were noted in this cohort, as 3% had a previous child ≤5 years death and 1.5% of women experienced a prior neonatal death between 24 and 100 days after birth.

Approximately 17% of women were considered a high-risk pregnancy and over 22% reported having any complication during the current pregnancy (Table 2). Any complications were defined as any adverse health outcome experienced during the pregnancy including but not limited to gestational diabetes, threat of miscarriage, hematoma, loss of amniotic fluid, abnormal bleeding, condylomatosis, and hemorrhage. Reasons for high-risk pregnancy included maternal age, threat of miscarriage, and placenta previa. One mother was considered high risk as the physician reported fetal myocarditis. Among all pregnancy complications, threat of miscarriage or stillbirth (9.6%) was reported most frequently.

Complications at the time of delivery were reported among 12% of participants: premature rupture of membranes (6%); breech birth (4%); and chorioamnionitis (1%). Any labor and delivery complications included but were not limited to neonatal asphyxia, UTI at the time of delivery, meconium aspiration, and neonatal obstetric trauma. Three mothers and neonates had to be transferred to a higher-level referent hospital for birth complications including fetal deterioration. Among those, one infant was stillborn due to prenatal asphyxia, but was revived at delivery and transferred to a higher clinical care hospital in the capital city. The median birthweight among neonates was 3070 g and median gestational age was 38 weeks. Any neonatal symptoms at the time of birth included but were not limited to ARDS, MAS, premature birth, suspected neonatal sepsis, cleft palate, and congenital malformation. Approximately 6% of neonates were born prematurely and 8% were low birthweight (<2500 g). Over 7% of neonates had APGAR < 9 at 1 min (range: 0–8) and 1.5% had APGAR < 9 at 5 min (range: 3–8). Among neonatal outcomes, 6% of neonates had acute respiratory distress syndrome (ARDS), 3% experienced meconium aspiration syndrome (MAS), and 6% were placed under a hood for respiration assistance.

**Figure 2:**
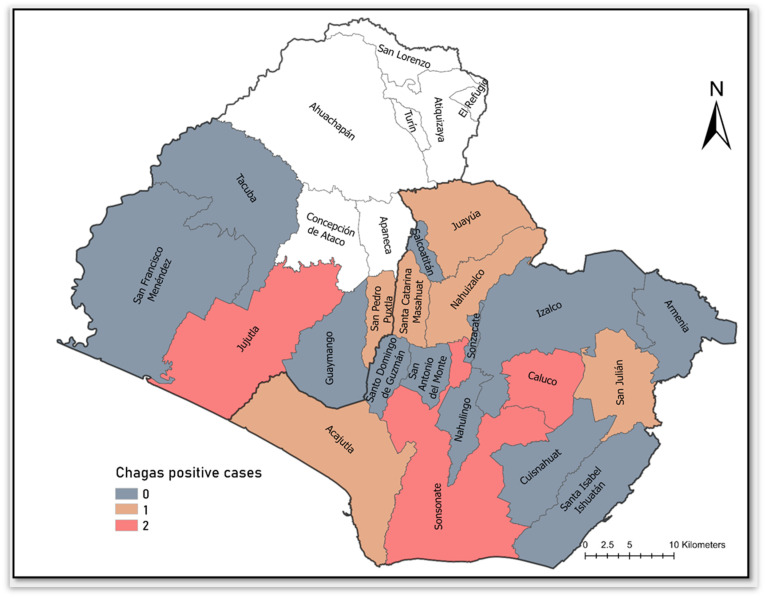
Map of *T. cruzi*-positive perinatal women from western El Salvador revealing that cases were mostly from Sonsonate department. The highest case count municipalities fell in Sonsonate and Caluco (Sonsonate department) and Jujutla (Ahuachapan department).

**Figure 3:**
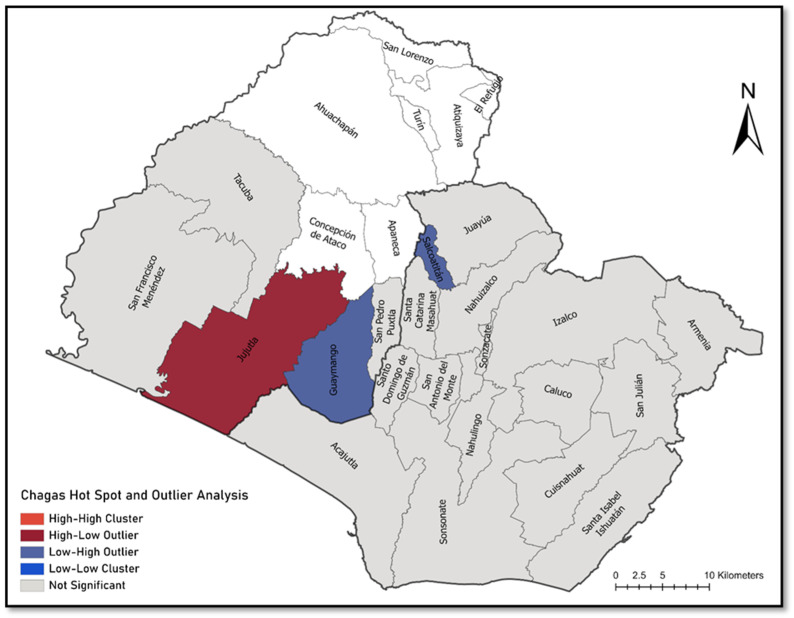
Cluster and outlier analysis of Chagas positive cases by municipality. High-low outliers indicate municipalities with comparatively higher *T. cruzi* positive prevalence from our participant population, surrounded by municipalities of lower prevalence. Low-high outliers indicate areas of lower *T. cruzi* positive prevalence from our study population, surrounded by municipalities with comparatively higher prevalence.

Among enrolled women, 12 women (6%) met disease diagnostic criteria for maternal *Trypanosoma cruzi* infection: 5 women with only dPCR positive result, 1 woman with a dPCR and ≥2 ELISA positive results, and 6 women with only ≥2 ELISA positive results. *T. cruzi*-positive mothers originated from seven municipalities in Sonsonate, and two municipalities in Ahuachapan, (Figure 2). Spatial statistics of clusters and outliers revealed Jujutla municipality as a high-low outlier—a municipality with comparatively higher *T. cruzi* positive prevalence than surrounding municipalities (Figure 3). The majority of Chagas disease positive participants were between 31 and 45 years of age (Table 1). Most women were homemakers, having no formal employment outside of the home. Over half of positive participants lived in significantly substandard housing and were of low socioeconomic status. One-third of participants reported a history of symptoms consistent with Chagas disease including foot inflammation, dyspnea or heart palpitations at rest, and difficulty climbing stairs; however, these symptoms can be common in late-stage pregnancy and baseline status recall was complicated by the >8 months of one’s pregnancy. No mothers had a known history of abnormal cardiac activity and ECGs were not performed at time of study enrollment.

Though the majority of positive participants had no knowledge of Chagas disease, one quarter of positive mothers had a friend or family member positive for the infection. The majority of positive women were familiar with the triatomine disease vector, and domestic exposure to the vector was reported among 16% of positive individuals. Indicators of potential *T. cruzi* transmission risk were infrequently reported, and included history of chagoma, severe insect bite, and recent intermittent fever. Only one positive individual lived in a house made of adobe walls and tin roofing. Neonatal outcomes of positive mothers included APGAR scores < 9 at 1- and 5-min, low birthweight, and premature birth (Table 2). Half of neonates born to positive mothers were sent to the NICU for ARDS, blood pressure, premature birth, MAS, premature rupture of membranes, or due to having a mother with diabetes.

Univariate analysis showed significant associations between *T. cruzi*-positive status and advanced maternal age (*p =* 0.002), lower maternal education level (*p* = 0.022), history of pregnancy complications (*p* = 0.051), and knowing someone with Chagas disease (*p =* 0.021) (Table 1). Univariate analysis also revealed significant relationships between *T. cruzi*-positive status and complications in the current pregnancy (*p =* 0.006), gestational diabetes (*p* = 0.019), and neonatal symptoms at the time of birth (*p =* 0.024). Maternal infection was further associated with neonatal complications including low 1-min and 5-min APGAR scores, NICU admission, and MAS, shown in Table 2. Results of multivariable logistic regression revealed maternal Chagas infection was associated with advanced maternal age (OR = 1.12, 95%CI: 1.03, 1.23) and knowing another individual who was positive for Chagas (OR = 8.31, 95%CI: 1.48, 40.24).

## 4. Discussion

A pilot study in Western El Salvador revealed 6% of perinatal women were Chagas disease positive at the time of childbirth—a higher rate than reported in the prior decade and contemporary rates in young children from the same region [2,19]. Infected mothers originated from nine municipalities—one municipality being spatially significant, highlighting the need for targeted maternal surveillance from these high-risk areas. Chagas disease was associated with negative maternal and neonatal outcomes among our population. As our population derived from highly vulnerable departments, further attention to Chagas disease in this region should be of high priority to improve maternal child health in this region.

Maternal *T. cruzi* infection was significantly associated with maternal age, likely due to increased lifetime triatomine vector exposure, consistent with prior studies [19,20,27], and having family members or acquaintances with Chagas disease. Familial clustering of cases is a common occurrence with Chagas disease due to overlapping vector-borne infection risk within households [28]. Nearly 18% of our study population reported knowledge of Chagas disease and 60% reported knowledge of triatomines. However, none of the participants correctly identified how the infection is transmitted, and knowing information about the infection was not a preventive factor in this perinatal population. Therefore, enhanced high-burdened community education campaigns could be beneficial in this region to improve personal understanding of infection risk. We found no association between substandard housing level and Chagas disease within our cohort. However, comparisons between groups were challenging as the majority of participants in this study were impoverished, experiencing similar poor socioeconomic status and substandard living conditions.

Pregnancy and neonatal outcomes in this study were consistent with prior studies of *T. cruzi*-infected pregnant women and neonatal infection [29]. Two neonates born to positive mothers were low birthweight, which has been associated with maternal Chagas disease positivity, regardless of neonatal infection [30]. Neonates born to positive mothers also had APGAR scores < 9 at 1 and 5 min, and premature birth. Half of neonates born to positive mothers in this study were sent to the NICU after PROM, MAS, or ARDS. Two neonates required breathing assistance via hood. Over half of congenital infections present asymptomatically, but low APGAR scores, ARDS, premature birth, and low birthweight were common in the absence of other symptoms among infected neonates [3,30]. Although we did not find any association with PROM, it has been previously associated with maternal and congenital infection due to trypomastigote invasion of the placental marginal zone and weaking of membranes surrounding the fetus [31]. None of the neonates were tested at the time of birth; therefore, congenital transmission events among this study population are unknown. The Ministry of Health and study team are in the process of neonatal follow up at ≥9 months to determine neonatal infection, as is standard [31].

We employed a highly sensitive molecular method, dPCR, which identified five positive individuals without serologic evidence of infection. These results could indicate detection of a recent infection or reactivation of a current chronic infection. Acute *T. cruzi* infection is common in this region [11,19], providing epidemiologic support for the five dPCR positive cases. Reactivation of chronic infection has an established link with immunosuppression, and evidence exists that heightened parasitemia of chronic and indeterminate infections may occur during pregnancy [32]. Pregnant women are immunologically vulnerable due to the natural shifts in immunity that occur during pregnancy, and reactivated cases could potentially be going undetected [32]. Sonsonate and Ahuachapan departments are considered Chagas disease endemic, with high national seroprevalence rates and home domiciliation of the disease vector [2]. Here, poverty exceeds national average, with 43.0% and 49.8% of households living below the poverty line, respectively [33]. The proportion of individuals living in substandard housing, as well as lacking potable water and sanitation services, also exceeds national averages in these departments, and >50% live in overcrowded homes [33]. These collective factors create ideal conditions for domestic triatomine infestation.

As slightly lower sensitivity of gold-standard serology assays has been shown within Central American populations, we employed all three serologic assays in parallel and used a further molecular assay to address this potential variation among our study population [34]. Digital PCR is an emerging technology that has been recently incorporated into diagnostic testing for human pathogens due to increased sensitivity, specificity, and the ability to detect low level parasitemia [35]. The use of dPCR in the field of human parasites is still emerging, and studies are limited in the context of Chagas disease. Digital droplet PCR was evaluated in one study for the detection Chagas disease in clinical blood samples from Colombia [35]. Although performance was not superior to qPCR in this study, the agreement between assays was 100% [24]. However, dPCR has shown superiority to other assays with several parasitic infections. Studies of *P. falciparum* found significantly higher sensitivity compared to qPCR, and increased precision and resilience to inhibitors was demonstrated when applied to *Cryptosporidium* [35]. To the best of our knowledge, this is the first study to employ dPCR in maternal samples, and these results give promise to this next-generation molecular diagnostic’s incorporation into future surveillance efforts.

Our spatial analysis found statistical clustering of maternal infections throughout the two western departments. These findings were similar to those of a previously published pediatric study in Sonsonate that found high case counts in in Nahuizalco and Santa Catarina Masahuat [19]. As clustering of disease cases frequently occurs with *T. cruzi* infection, results of this study support prior evidence that ongoing vector-borne transmission is still occurring and a portion of pediatric cases in this region of El Salvador could possibly be contributed to congenital infection. A recent publication of *T. dimidiata* dispersion found a 10% overall *T. cruzi* infection rate within the disease vector and an elevated infection rate in the departments of Ahuachapan and Sonsonate [36]. The study further found >34% of surveyed houses had triatomine domiciliation. Adobe houses were most highly colonized by triatomines followed by mud and brick [36]. Substandard living conditions and socioeconomic limitations in certain parts of the western departments create risk for exposure to infected insects. The reported rate of triatomine home infestation in these departments was also approximately 10% in 2007 [37], suggesting sustained fecundity and vector–human contact in this region. Future vector and housing improvement efforts should target these endemic geospatial clusters.

Congenital infections, such as *T. cruzi*, are not uncommon in El Salvador and mandatory prenatal HIV and syphilis screening is nationally implemented. In 2020, the Salvadoran Ministry of Health reported 0.05% positive syphilis tests (42/85,080) and 0.04% (34/85,080) positive HIV tests among prenatal care screenings [38]. In contrast, 6.1% of pregnant women from this small pilot study were *T. cruzi* infected, yet this disease remains neglected in routine prenatal testing. Similar to prior studies performed one decade earlier, *T. cruzi* seroprevalence is consistently higher among pregnant women (3.6%) than blood donors (1.7%), making this vulnerable group disproportionately at risk of severe disease outcomes [20,39]. Prenatal Chagas disease testing of pregnant women and women of child-bearing age must be mandated in high-risk departments. The Salvadoran health care system guidelines for prenatal care recommend four prenatal visits for pregnant women [40], and prenatal care coverage has been high across the country since maternal–child health law reforms in 2010 [41]. Prenatal care in this rural low-income region was higher than expected, presenting the possibility of further education and intervention in this region. Nearly 96% of the study participants received at least one prenatal care visit in all three trimesters, though the total number of visits was not captured. Due to the increased perinatal care coverage propagated by the recent Ley Nacer con Cariño, screening for Chagas disease could have wide-reaching implications in early identification of congenital cases, access to treatment for infected neonates, and the overall better understanding of congenital infection in this country.

This study was limited by sample size (n = 198 participants) and geographically limited to two western departments. Though these departments both fall under a high index of multidimensional poverty [33], future works should encompass further departments with low socioeconomic status and substandard housing, both being well-established risk factors for Chagas disease infection [14,42,43]. Infant testing results were unavailable for the current study, limiting our knowledge of vertical transmission in this cohort; however, the Ministry of Health is actively performing this testing, ensuring proper treatment of both mothers and any subsequently infected infants. This study leveraged a highly sensitive molecular method to detect Chagas disease infection, which nearly doubled the detection rate, highlighting the value of dPCR in this diagnostic context. Future studies should employ this platform to evaluate the utility of dPCR in the context of Chagas disease, as only 2% of infections are identified in the acute phase, and early identification of cases is critical for prognosis [26,44]. DPCR could serve as a useful tool in the early identification of acute cases, as well as cases of reactivation during pregnancy, to provide clinical suspicion for testing and follow-up of mothers and neonates.

## 5. Conclusions

Although the prevalence of Chagas disease in El Salvador has potentially decreased post-*Rhodnius prolixus* elimination (a highly domesticated vector species [20]), this study presents evidence that transmission is still ongoing. The high rate of Chagas disease infection compared to that of other infectious pathogens included in prenatal care screening warrants further, larger-scale surveillance in high-risk areas of El Salvador. Screening of pregnant women at the time of labor and delivery is particularly useful in identifying congenital cases of infection, administering treatment, and testing other at-risk household members. Post-delivery treatment of positive mothers could further prevent the potential risk for vertical transmission in future pregnancies. DPCR is an emerging technology for direct quantification of parasitic infections which requires further evaluation across geographies, but which could be a promising tool for the early detection of cases and the global enhancement of Chagas disease surveillance.

## Figures and Tables

**Table 1:** Perinatal Salvadoran women primarily lived in low socioeconomic conditions and Chagas disease positivity was associated with several demographic factors.

	% (N) Total Study Population (*n* = 198)	% (N) Chagas (+) Mothers (*n* = 12)	% (N) Chagas (−) Mothers (*n* = 186)	* *p*-Value, Fisher’s Exact Statistic
Department				0.415
Ahuachapan	16.2% (32)	25.0% (3)	15.6% (29)	
Sonsonate	83.8% (166)	75.0% (9)	84.4% (157)	
Age Group				0.002
<18 years	7.1% (14)	8.3% (1)	7.0% (13)	
18–25 years	43.9% (87)	8.3% (1)	46.2% (86)	
26–30 years	21.7% (43)	8.3% (1)	22.6% (42)	
31–45 years	27.3% (54)	75.0% (9)	24.2% (45)	
History of Miscarriage				0.370
No	89.4% (177)	83.3% (10)	89.8% (167)	
Yes	10.6% (21)	16.7% (2)	10.2% (19)	
High Risk Pregnancy (current)				0.119
No	83.3% (165)	66.7% (8)	84.4% (157)	
Yes	16.7% (33)	33.3% (4)	15.6% (29)	
Prior pregnancy complications (any)				0.051
No	92.4% (183)	75.0% (9)	92.5% (172)	
Yes	7.6% (15)	25.0% (3)	6.5% (12)	
Mother’s education level				0.022
≤6th grade	43.4% (86)	58.3% (7)	42.5% (79)	
7th grade–11th grade	25.8% (51)	16.7% (2)	26.3% (49)	
High school	28.3% (56)	8.3% (1)	29.6% (55)	
University or Graduate	2.5% (5)	16.7% (2)	1.6% (3)	
Number of prior children *				0.096
none	36.9% (73)	33.3% (4)	37.3% (69)	
1–3	59.1% (117)	50.0% (6)	60.0% (111)	
4+	3.5% (7)	16.7% (2)	2.7% (5)	
Knowledge of Chagas Disease				1.0
No	82.3% (163)	83.3% (10)	82.3% (153)	
Yes	17.7% (35)	16.7% (2)	17.7% (33)	
Knowledge of triatomines *				0.349
No	39.4% (78)	50.0% (6)	38.9% (72)	
Yes	60.1% (119)	41.6% (5)	61.6% (114)	
Knowing someone with Chagas disease				0.021
No	94.4% (187)	75.0% (9)	95.7% (178)	
Yes	5.6% (11)	25.0% (3)	4.3% (8)	
Have electricity in the home *				1.0
No	7.6% (15)	0% (0)	8.1% (15)	
Yes	91.9% (182)	91.7% (11)	91.9% (171)	
Significant substandard housing level *				0.089
No	69.7% (138)	41.6% (5)	71.5% (133)	
Yes	29.8% (59)	50.0% (6)	28.5% (53)	
Total number in household *				1.0
<3	19.7% (39)	16.7% (2)	20.0% (37)	
4–6	67.7% (134)	75.0% (9)	67.6% (125)	
7+	12.1% (24)	8.3% (1)	12.4% (23)	

* Indicates one participant did not answer this question and a different denominator was present.

**Table 2:** Chagas disease positivity was associated with complications during pregnancy and neonatal symptoms at the time of birth.

	% (N) Total Study Population (*n* = 198)	% (N) Chagas (+) Mothers (*n* = 12)	% (N) Chagas (−) Mothers (*n* = 186)	*p*-Value, Fisher’s Exact Statistic
Pregnancy Complications				
Any complications	22.2% (44)	58.3% (7)	19.9% (37)	0.006
Gestational diabetes	2.0% (4)	16.7% (2)	1.1% (2)	0.019
Threat of miscarriage	9.6% (19)	8.3% (1)	9.7% (18)	1.0
Placenta previa or acreta	2.0% (4)	0% (0)	2.2% (4)	1.0
Cesarean section (c-section)	55.1% (109)	100.0% (12)	52.2% (97)	0.001
Breech birth	4.0% (8)	0% (0)	4.3% (8)	1.0
Urinary tract infection (UTI) during pregnancy	2.5% (5)	0% (0)	2.7% (5)	1.0
Labor and Delivery Complications				
Any	11.6% (23)	25.0% (3)	10.7% (20)	0.159
Premature rupture of membranes (PROM)	5.6% (11)	8.3% (1)	5.4% (10)	0.514
Neonatal symptoms at birth				
Any symptom	10.1% (20)	33.3% (4)	8.6% (16)	0.024
Low birthweight	7.6% (15)	16.7% (2)	7.0% (13)	0.234
Apgar 1 min < 9	7.1% (14)	33.3% (4)	5.4% (10)	0.006
Apgar 5 min < 9	1.5% (3)	16.7% (2)	0.5% (1)	0.010
Admitted to neonatal intensive care (NICU)	20.2% (40)	50.0% (6)	18.3% (34)	0.020
Premature birth	5.6% (11)	8.3% (1)	5.4% (10)	0.514
Meconium aspiration syndrome (MAS)	2.5% (5)	16.7% (2)	1.6% (3)	0.032
Acute respiratory distress syndrome (ARDS)	5.6% (11)	16.7% (2)	4.8% (9)	0.141
Respiratory assistance (hood)	5.6% (11)	16.7% (2)	4.8% (9)	0.141

## Data Availability

The data presented in this study are available on request from the corresponding author. The data are not publicly available due to concern for loss in Spanish–English translation and patient confidentiality issues.

## Supplementary Materials

The following supporting information can be downloaded at: https://www.mdpi.com/article/10.3390/tropicalmed8040233/s1, Table S1: Targeted sequences used in digital PCR for molecular *T. cruzi* detection, Supplementary file: Congenital Chagas disease participant questionnaire (in original language).
