# Supplementary material for: Chagas Disease Maternal Seroprevalence and Maternal–Fetal Health Outcomes in a Parturition Cohort in Western El Salvador
Source: Trop Med Infect Dis. 2023 Apr 20;8(4):233. doi: 10.3390/tropicalmed8040233 (PMC10146685; doi:10.3390/tropicalmed8040233)
Supplement: Supplementary file 1 [file tropicalmed-08-00233-s001.zip › tropicalmed-2338585-supplementary.pdf]

Supplementary Table S1: Targeted sequences used in digital PCR for molecular *T. cruzi* detection.

Oligonucleotide	Sequence 5′-3′
**Cruzi 1**	**ASTC-GGCT-GATC-GTTT-TCGA**
**Cruzi 2**	**AATT-CCTC-CAAG-CAGC-GGAT-A**
**Cruzi 3**	**FAM-CACACACTGGACACCAA-NFQ-MGB**
**HsRPP30- Forward**	**AGATTTGGACCTGCGAGCG**
**HsRPP30- Reverse**	**GAGCGGCTGTCTCCACAAGT**
**HsRPP30-Probe**	**Hex-TTCTGACCTGAAGGCTCTGCGCG-Qsy-7**

Número de identificación del estudio:__________

<u>Prevalencia y Factores de Riesgo de la Enfermedad de Chagas Congénito en mujeres embarazadas en zonas Endémicas en El Salvador.</u>

Gracias por su tiempo y participación. La meta de nuestra investigación es poder comprender con qué frecuencia las mujeres en estado de embarazo transmiten la Enfermedad de Chagas a sus hijos y cuáles son los factores de riesgo por infección entre mujeres gestación. Solo debe responder las preguntas que usted elija.

Fecha de la Entrevista: ______________________ **Entrevistador**: ________________________________

Nombre completo de la Mamá: ____

Fecha de nacimiento: ______________________ **Ciudad y país de nacimiento:** _____________________

Dirección: ________________________________ **Departamento:** ________________________________

Municipio: _______________________________ **Cantón:** _______________________________________

Caserío: __________________________________**Número de teléfono:** ____________________________

Correo electrónico: ____________________________

Nombre completo del Papá:____

Número de teléfono: ____

Dirección (si es diferente de la anterior): ____

Correo electrónico: ____

Otro número de contacto (si tiene):

Contacto (nombre y número de teléfono): ____

Relación con usted: ____

<u>Resultados de indicadores de preeclampsia</u>

Presión: _______ / __________

Tiras de prueba de orina por proteinuria : *Positivo* *Negativo*

Tiras de prueba de orina por glucosuria : *Positivo* *Negativo*

Notas:

<u>Sección 1: Embarazo Actual /Historia Clínica y Demográfico</u>

1.) ¿Cuál es la fecha de nacimiento de la mamá (día, mes, año)? ____________________________________

2.) ¿Cuál es la estatura de la mamá?________________________________

3.) ¿Cuál es el peso de la mamá? __________________________________

4.) ¿Cuál es su probable fecha de parto? ____

5.) ¿Cuál es su probable lugar donde dara a luz?____

5.a) *Dirección, Municipio, Cantón, Caserío* ____

6.) ¿Planeas amamantar al bebé? ____

6.a) *¿por cuánto tiempo?* ____

7.) ¿Cuántas veces ha estado embarazada? ___________________________

8.) ¿Cuántos bebes ha tenido? _______________________________________

9.) ¿Has estado embarazada en los últimos 18 meses? *Si* *No*

10.) Control prenatal en: 1st Trimestre: _______ 2nd Trimestre: ___________ 3rd Trimestre: _________

11.) ¿Ha tenido COVID en algún momento durante su embarazo? ____________________________________

12.) ¿Su embarazo es de alto riesgo? *Sí* *No*

13.) ¿Tiene complicaciones en el embarazo? *Sí* *No*

13a.) *Si , ¿cuáles?*

preeclampsia *hipertensión gestacional* *diabetes prenatal* *asma*

uso de tabaco *uso de otras drogas o alcohol* *restricción intrauterina crecimiento*

presión arterial *recuento de plaquetas* *proteinuria* *cardiomiopatía*

obesidad *insuficiencia de cérvix* *depresión* *inflamación de las glándulas*

*Otro:*____

14.) ¿Ha tenido alguna complicación en embarazos anteriores? *Sí* *No*

14a.) *Si es así, ¿qué complicaciones?*____

15.) ¿Alguna vez ha recibido una transfusión sanguínea? *Sí* *No*

15a.) *De responder sí, ¿en qué año y lugar (ciudad/estado/país y cuantas veces)?*________________

____

16.) ¿Alguna vez ha recibido algún trasplante de tejidos u órganos? *Sí* *No*

16a.) *De responder sí, ¿dónde y cuándo?* ____

17.) ¿Con que frecuencia experimenta inflamación o hinchazón en los pies?

rara vez *algunas veces* *nunca*

18.) ¿Con frecuencia experimenta dificultad para respirar cuando se acuesta?

rara vez *algunas veces* *nunca*

19.) ¿Con frecuencia experimenta problemas para subir escaleras?

rara vez *algunas veces* *nunca*

20.) ¿Con frecuencia experimenta que su corazón se acelera cuando está descansando?

rara vez *algunas veces* *nunca*

21.) ¿Alguna vez un doctor le ha dicho que el latido de su corazón no es normal? *Si* *No*

22.) ¿Cuándo fue la última vez que tu fue a la clínica / médico? ______________________________________

23.) ¿Cuándo fue la última vez que tu fue al dentista?____

24.) ¿Cuántas veces se enfermó el año pasado (visitas que realizo al médico, etc.)?

pocas veces ninguna vez Describir: ________________________________

25.) ¿Cuál es su estado civil?

Soltera *Casada* *Acompañada* *Divorciada* *Otros*

26.) ¿Cuál es el nivel de educación de la mamá?

Ninguno *Básico* *Tercer Ciclo* *Bachillerato* *Universitario*

27.) ¿Cuál es el nivel de educación del papá?

Ninguno *Básico* *Tercer Ciclo* *Bachillerato* *Universitario*

28.) ¿Cuál es su ocupación actual de la mamá

Estudiante *Empleada* *Ama de Casa* *Otro*_____________________

29.) ¿Cuál es su ocupación actual del papá?

Estudiante *Agricultor* *Obrero* *Empleado* *Otro*________________

30.) ¿Cuántos hijos tiene usted? ____________

31.) ¿Cuántos de sus hijos asisten a la escuela? ______________

32.) ¿Con qué frecuencia te preocupas al saber de dónde vendrá los alimentos en el siguiente tiempo de comida para tu familia?

Nunca *A Veces* *A Menudo* *Siempre*

33.) En el último mes, ¿Cuántos Días ha faltado Alimentos para su familia? ______ Días.

34.) ¿Normalmente come *los tres tiempos de* comida al día? *Sí* *No* *A veces*

35.) En el último mes, ¿Su familia comió los mismos alimentos todos los días? *Sí* *No*

35a.) *¿Qué comía o come normalmente su familia?* __________________________________

36.) En las últimas dos semanas, ¿con qué frecuencia se ha sentido estresado o preocupado?

Casi Siempre *Algunas Veces* *Nunca*

36a.) *¿Por qué estas estresado?*

Alimentación *Inseguridad* *Salud*

Dinero *Violencia/Crimen* *Otros:* ______________________________

37.) ¿Te Preocupa la seguridad / Inseguridad de su caserío? *Sí* *No*

37a.) *¿Cuáles son sus preocupaciones específicas?* ____

____

38.) ¿Preocupa de la seguridad en casa? *Sí* *No*

38a.) *¿Cuáles son sus preocupaciones específicas?* ____

39.) A lo largo de su vida, ¿Ha experimentado alguna vez violencia con alguna pareja o esposo?

Sí *No*

39a.) *¿Cuántas veces lo experimentado esto?*

1-2 veces *5-10 veces* *Más de 10 veces*

Sección 2: Descripción de Familia, Hogar y Vivienda

40.) ¿Cuántas personas de cada grupo de edades viven en su casa?

¿niños <5 años?__________________ ¿adultos mayores de 40 años?______

¿niños 5-17 años?_________________ ¿ embarazadas?_________________

¿adultos jóvenes 18-40 años?________ ¿ personas en total?______________

41.) El año pasado alguien de su familia ha experimentado:

41a.) ¿ Roncha grande en el cuerpo que dura más de una semana? *Sí* *No*

41b.) ¿ Erupción manchada en el cuerpo que dura más de una semana? *Sí* *No*

41c.) ¿Una picadura de insecto que produjo una costra notablemente grave? *Sí* *No*

41d.) ¿Una Fiebre Intermitente? *Sí* *No*

42.) ¿Conocen la Enfermedad de Chagas? *Sí* *No*

42a.) ¿ *Que conoce de la enfermedad de Chagas?* ___________________________________

43.) ¿Conoces a alguna persona que ha tenido o tiene la Enfermedad de Chagas?

43a.) *¿Quién?*____

44.) ¿Conoces cómo se enferma una persona de la Enfermedad de Chagas? *Sí* *No*

44a.) ¿ *Como se transmite la enfermedad de Chagas?*_____________________________

45.) ¿Si usted se da cuenta que su hijo tiene la Enfermedad de Chagas, buscaría ponerlo en tratamiento?

Sí *No*

46.) ¿Qué enfermedades padecen sus familiares que habitan en su mismo hogar?___________________

47.) ¿Qué tipo de paredes tiene la casa?

adobe *bloque* *ladrillo* *bajareque*

madera *piso de tierra* *bahareque* *sin pared*

otro: ¿cuál? _________________

48.) ¿Qué tipo de repello tiene la casa?

repello con cemento *repello con lodo/con tierra*

parcialmente repellado *sin repello*

49.) ¿Qué tipo de techo tiene la casa?

paja *palma* *lamina* *duralita*

zinc *madera* *Tejado* *otro: ¿cuál?*_______________

50.) ¿Qué tipo de piso tiene la casa?

tierra *cemento* *baldosa* *madera*

piso cerámico *otro: ¿cuál?_________________*

51.) ¿Cuáles estructures adiciónales tiene la casa?

gallinero *bodega* *porqueriza* *horno/cocina de leña*

conejera *trapiche* *ninguno* *otro: ¿cuál?_________________*

52.) ¿Qué tipo de agua usa en su casa?

potable *de pozo* *nacimiento* *río* *otro: ¿cuál?_______________*

53.) ¿Qué tipo de baño tienen?

de lavar *letrina* *no tengo* *otro: ¿cuál?_________________*

54.) ¿Presencia de grietas en las paredes? *Sí* *No*

55.) ¿Cuántas focos tiene al interior de la casa?_____________________ alrededor de la casa?________

56.) ¿De qué vive económicamente la familia (agricultura, comercio, empresa, remesa, otro)?

____

57.) ¿Cuántas camas hay en la casa?____

58.) ¿Están las paredes del dormitorio repellada? *Sí* *No*

59.) ¿Hay ventana en la habitación? *Sí* *No*

60.) ¿Tiene energía eléctrica? *Sí* *No*

61.) ¿Qué tipo de cocina posee?

gas *leña* *ambos* *otro: ¿cuál?_________________*

62.) ¿Tienes refrigerador que funcione en la casa? *Sí* *No*

62a.) *¿Dónde guardas tu comida?* ____

Sección 3: Vectores

63.) ¿Qué tipo de insectos vio adentro o cerca de su casa?

mosquitos *garrapatas* *pulgas* *chinches*

arañas *mosca* *ninguna* *otro: ¿cuál?* _________________

64.) ¿Alguna vez ha visto insectos llenos de sangre adentro o cerca de su casa? *Sí* *No*

64a.) *¿Si dicen que sí, alguna vez los vio en animales o en un miembro de su familia?* *Sí* *No*

64b.) *¿Cuáles insectos?*____

65.) ¿Si usted observara un insecto en su vivienda que hace?

ignorarlo *utiliza algún objeto para matar el insecto*

lo elimina manualmente *lo captura y lo deposita en la basura*

lo quema *recolecta el insecto y reporta su presencia a un ente encargado*

otro: ¿otra acción ?_______________

66.) ¿Conocen las chinches?: *Si* *No*

66a.) *¿En el último año han visto chinches dentro o alrededor de su casa?:* *Sí* *No*

66b.) *¿En qué sitio ha visto estos insectos?:*

Intradomicilio: Espacio donde duerme la gente, parte interna de la casa

cocina *dormitorio* *sala* *comedor*

pasillos *baños* *corredor* *otro: ¿cuál?_______________*

Peridomicilio: alrededor de la casa, fuera del techo de la casa

gallinero *bodega* *monte* *material de construcción*

pante de leña *en indos de aves* *cactus* *otro: ¿cuál?_______________*

Extra-domicilio: Área por fuera del ámbito de actividades cotidianas de los habitantes

cultivos *bosque* *otro: ¿cuál?_______________*

67.) ¿En el último año han picado las chinches a alguno de la familia? *Sí* *No*

68.) ¿Sintió usted alguna reacción adversa (alérgico)? *Sí* *No*

68a.) *¿Si dicen que sí: Por favor responda ¿Qué tipo de reacción negativa sintió?*

irritación de los ojos *picazón en la piel* *asfixia o dificultad para respirar*

mareo *vomito* *otro: ¿cuál?_______________*

69.) ¿Qué tipo de animales tienen?

ninguno *perros* *gallinas* *cerdos* *vacas*

caballos *gatos* *aves domésticas* *conejos domésticos*

otro: ¿cuál?_______________

70.) ¿Tienen animales adentro de la casa? *Sí* *No*

70a.)*¿Qué tipo?*____

71.) ¿Qué tipo de los animales silvestres ha visto cerca o dentro de su vivienda?

ninguno *tacuazín* *roedores* *murciélagos*

palomas *conejos silvestres* *cotuza* *venado*

cusuco *gato montés* *otro: ¿cuál?_______________*

71a.) *¿En cuáles de los siguientes ambientes los ha visto?*

dentro de la casa *alrededor de la casa* *en el bosque* *otro: ¿cuál?_____________*

72.) ¿En el último año ha recibido alguna visita de la unidad de salud? *Sí* *No*

72ª.) *¿cuál fue el motivo de la visita?*

no sabe *encuestar a un habitante de la vivienda* *fumigar*

inspeccionar la vivienda *vacunación* *hacer búsqueda de insectos*

otro: ¿cuál?_____________

73.) ¿Usted usa mosquitero? *Sí* *No*

73a.) *¿cuántos tiene?* ____

74.) ¿Quién de su familia suele dormir bajo el mosquitero?____

75.) ¿Cuánto tiempo llevas usando mosquitero?____

76.) ¿Tiene algún otro método para controlar insectos dentro de su casa? *Sí* *No*

76a.) *Describa el método:*

____

Por Equipo del Estudio: Sección 4: Trabajo de Parto, Parto y Resultados de Salud Neonata

77.) Número de identificación del bebe en el estudio: ______________________________________

78.) Numero identificación del hospital donde nació él bebe: ________________________________

79.) Nombre completo del bebe: ____

80.) Fecha de Nacimiento del bebe: ____

81.) Sexo del bebe: *masculino* *femenino*

82.) Edad gestacional: ______Semanas ______Días

83.) Tipo de parto: *natural* *cesárea* *otro: ¿cuál?* _______________

84.) Peso del bebé: _____ lb ____ oz

85.) Talla del bebé: ____________ cm

86.) APGAR de neonatas(os) de minuto 1: ________

87.) APGAR de neonatas(os) de minuto 5: ________

88.) Resultado(s) del nacimiento: *parto prematuro* *pérdida fetal* *otro: ¿cuál?* _____________

89.) Complicaciones al momento de labor de parto/parto?: *Sí* *No*

asfixia perinatal *sangrado excesivo* *placentitis/villitis*

otro: ¿cuál? _____________

90.) ¿Recibió esteroides dentro de las 24 horas posteriores de parto: *Sí* *No*

91.) ¿Se transfirió el(los) neonato(s) a la unidad de cuidados intensivos neonatal? *Sí* *No*

91a) *Si dice si, cuantos días estuvo en la unidad*: ____

92.) Fue él bebe a término (>37 semanas): *Sí* *No*

93.) ¿Es el bebe positivo a VIH? *Sí* *No*

¿A sífilis? *Sí* *No*

94.) Experimentó él bebe algunos síntomas en la primera semana de vida: *Sí* *No*

fiebre *hepatoesplenomegalia* *anemia* *meningitis*

miocarditis *síndrome de dificultad respiratoria* *hidrocefalia* *trombocitopenia*

mala alimentación *erupción* *convulsiones* *retinitis*

*otro: ¿cuál?*____

95.) Tiene complicaciones o diagnóstico dentro del primer mes de vida: *Sí* *No*

fiebre *erupción* *el síndrome de dificultad respiratoria aguda (ARDS)*

neumonía *apnea* *pérdida de la visión*

retraso en el desarrollo *conducto arterioso persistente* *Displasia broncopulmonar (BPD)*

*otro: ¿cuál?*____

96.) Necesito soporte de oxígeno o ventilador mecánico durante el primer mes de vida: *Sí* *No*

Cánula nasal *CPAP* *ventilación mecánica* *OMEC*

Nota(s) adicionales: ____

Para el equipo del Estudio: Sección 5 Resultados de Pruebas

Resultados de las pruebas por Chagas:

97. Prueba de Weiner Chagatest: *Positivo* *Negativo*

98. Prueba de ensayo rápido: *Positivo* *Negativo* *No administrada*

99. Prueba de qPCR: *Positivo* *Negativo*

99ª.Carga de parásitos: _____________ *equivalentes de parásitos por mL (por qPCR)*
